# Quantitative Magnetization Transfer in Monitoring Glioblastoma (GBM) Response to Therapy

**DOI:** 10.1038/s41598-018-20624-6

**Published:** 2018-02-06

**Authors:** Hatef Mehrabian, Sten Myrehaug, Hany Soliman, Arjun Sahgal, Greg J. Stanisz

**Affiliations:** 1 Medical Biophysics, University of Toronto, Toronto, Ontario, Canada; 2 Physical Sciences, Sunnybrook Research Institute, Toronto, Ontario, Canada; 3 Radiation Oncology, Sunnybrook Health Sciences Centre, Toronto, Ontario, Canada; 4 Department of Radiation Oncology, University of Toronto, Toronto, Ontario, Canada; 5 Department of Neurosurgery and Pediatric Neurosurgery, Medical University, Lublin, Poland

## Abstract

Quantitative magnetization transfer (qMT) was used as a biomarker to monitor glioblastoma (GBM) response to chemo-radiation and identify the earliest time-point qMT could differentiate progressors from non-progressors. Nineteen GBM patients were recruited and MRI-scanned before (Day_0_), two weeks (Day_14_), and four weeks (Day_28_) into the treatment, and one month after the end of the treatment (Day_70_). Comprehensive qMT data was acquired, and a two-pool MT model was fit to the data. Response was determined at 3–8 months following the end of chemo-radiation. The amount of magnetization transfer (${\bf{R}}{{\bf{M}}}_{{\bf{0b}}}/{{\bf{R}}}_{{\bf{a}}}$) was significantly lower in GBM compared to normal appearing white matter (p < 0.001). Statistically significant difference was observed in ${\bf{R}}{{\bf{M}}}_{{\bf{0b}}}/{{\bf{R}}}_{{\bf{a}}}$ at Day_0_ between non-progressors (1.06 ± 0.24) and progressors (1.64 ± 0.48), with p = 0.006. Changes in several qMT parameters between Day_14_ and Day_0_ were able to differentiate the two cohorts with ${\bf{R}}{{\bf{M}}}_{{\bf{0b}}}/{{\bf{R}}}_{{\bf{a}}}$ providing the best separation (relative ${\bf{R}}{{\bf{M}}}_{{\bf{0b}}}/{{\bf{R}}}_{{\bf{a}},{\bf{Non}}-{\bf{progressor}}}$ = 1.34 ± 0.21, relative ${\bf{R}}{{\bf{M}}}_{{\bf{0b}}}/{{\bf{R}}}_{{\bf{a}},{\bf{progressor}}}$ = 1.07 ± 0.08, p = 0.031). Thus, qMT characteristics of GBM are more sensitive to treatment effects compared to clinically used metrics. qMT could assess tumor aggressiveness and identify early progressors even before the treatment. Changes in qMT parameters within the first 14 days of the treatment were capable of separating early progressors from non-progressors, making qMT a promising biomarker to guide adaptive radiotherapy for GBM.

## Introduction

Glioblastoma (GBM) is the most common malignant primary brain tumor in adults^1^ with a median survival rate of 15–18 months for newly diagnosed patients^2,3^. As a result of this prognosis, patients are treated with a combination of surgical resection, radiotherapy and chemotherapy^4–6^. Current response evaluation criteria rely on changes in tumor size^7^ which may take weeks or month to occur, by which time the therapeutic window is often lost. A non-invasive biomarker capable of determining response before (by characterizing tumor aggressiveness) or during the early phases of the treatment could have significant clinical utility. The treatment for patients with progressive tumors could be changed or adjusted, and for non-progressors a personalized radiotherapy approach could be used. As radiotherapy advances into the era of daily MRI guidance^8^, the ability to understand changes in brain tumors during the course of a 6-week chemo-radiation treatment is a new area of investigation, and one where novel methods that are not contrast based are desperately needed.

Imaging biomarkers are routinely used in assessing GBM response to treatment. The response evaluation in neuro-oncology (RANO) criteria combines the information from post-Gd T_1_-weighted (T_1w_)-MRI and T_2_-weighted FLAIR to evaluate response (mainly through measuring change in tumor size)^7^. More advanced techniques have also been used such as dynamic contrast enhanced (DCE)-MRI^9^, dynamic susceptibility enhanced (DSC)-MRI^9^, diffusion weighted-MRI (apparent diffusion coefficient)^10^, chemical exchange saturation transfer (CEST)^11^, magnetic resonance microscopy (MRS)^12^ in response evaluation. These advanced biomarkers have been studied at one to three months post-therapy, which is the accepted standard time-point for response evaluation in clinical practice. However, their potential in evaluating response during or early after treatment is yet to be investigated.

Magnetization transfer (MT)-MRI is a contrast mechanism that is sensitive to concentration of macromolecular protons and their exchange with free water protons. Quantitative MT (qMT) enables measuring the characteristics of the macromolecular protons including bound proton fraction, relaxation times of the bound and free water pools, as well as the exchange rate between the two pools. These tumor characteristics are more sensitive to treatment effect such as apoptosis^13^ and Temozolomide-induced pH changes^14^ (through altered exchange rate), and reflect treatment-induced changes in the tumor much earlier (as early as 48 hours)^13^ than clinically used imaging metrics (which are based on changes in linear dimensions of the tumor).

Semi-quantitative MT and qMT has been previously applied to multiple sclerosis^15,16^ and Alzheimer’s disease^17,18^ where they were able to correctly identify disease presence. qMT was also used in characterizing the changes in brain tissue in HIV patient where it showed a significant reduction in MT parameter compared to healthy controls^19^. In cancer, and in particular in GBM, there have been very few studies that have investigated qMT properties of the tumor^20,21^. Tozer *et al*.^20^ calculated qMT parameters in GBM in eight GBM patients and reported that the tumor has significantly decreased MT compared to white matter and gray matter. Similar results were also obtained by Xu *et al*.^22^ and Underhill *et al*.^23^ in glioma models in rats. More recently, Arlinghaus *et al*.^24^ investigated and showed the reproducibility of qMT parameters in healthy breast tissue as a first step to applying it to breast cancer evaluation in clinic. Moreover, Li *et al*.^25^ used a more complex four-pool MT model for brain tissue and concluded that a two-pool model is sufficient for accurate characterization of MT properties of brain tissue. To the best of our knowledge, there has been no study that looked into the potential of qMT biomarkers in assessing GBM response to treatment or its progression during the treatment.

The current study investigates the changes in the qMT parameters in GBM over the course of its 6-weeks of chemo-radiation treatment (through MRI-scanning patients at multiple time points during treatment) and attempts to find the earliest time point at which qMT could separate early progressors from non-progressors. Being able to assess therapeutic response of GBM at early phases of the treatment or even before the start of the treatment enables the oncologists to tailor the treatment to the individual patients, avoid ineffective treatments, and potentially improve outcome.

## Results

Three out of the total 19 recruited patients were removed from analysis due to significant imaging artifacts (one case), patient requested termination of the baseline scan (one case), R_1_/B_1_ mapping data was missing (one case). The remaining 16 patients were classified by the blinded oncologist into non-progressors (10 patients) and progressors (6 patients). A few of the patients did not complete all four scans and therefore there were the following number of patients as each time point:

Day_0_: 10 Non-progressors, 6 Progressors

Day_14_: 10 Non-progressors, 4 Progressors

Day_28_: 8 Non-progressors, 3 Progressors

Day_70_: 10 Non-progressors, 3 Progressors

**Figure 1:**
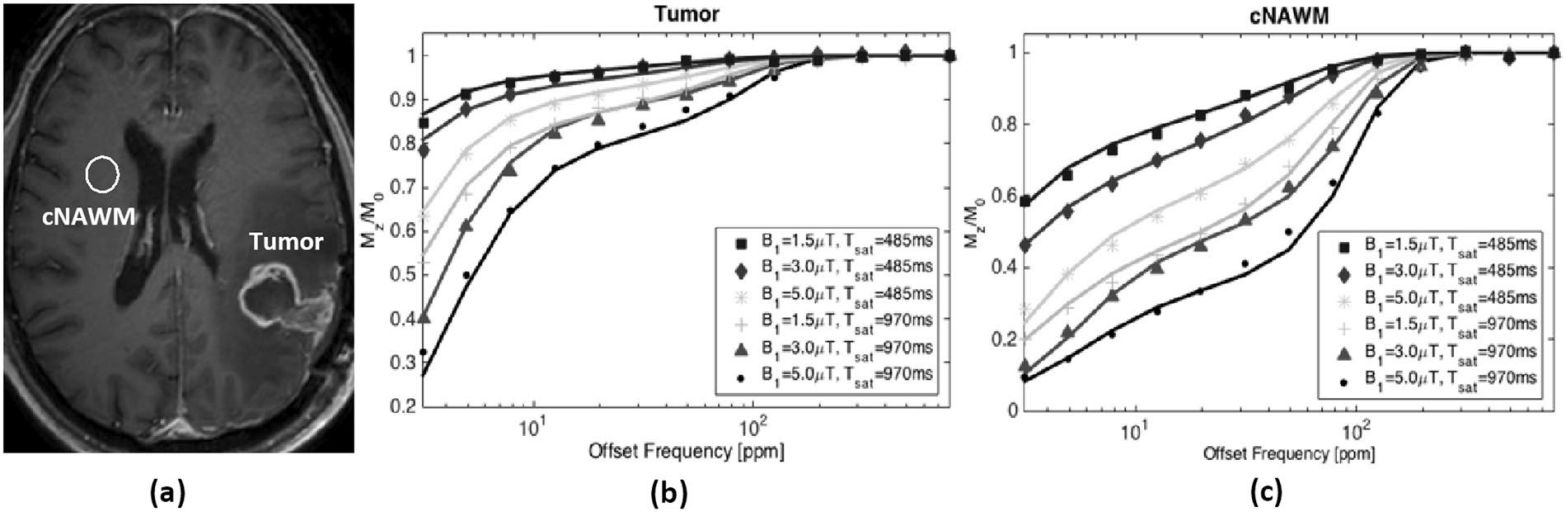
(**a**) Post-Gd T_1w_ image of a representative GBM patient at Day_0_, showing the tumor and contralateral normal appearing white matter (cNAWM) ROIs. The six acquired MT spectrums (dotted lines) and the two-pool MT fit (solid lines) for (**b**) Tumor and (**c**) cNAWM (spectra are averaged over ROIs) are shown.

The tumor volume was determined as the area encompassed by the enhancing tumor rim on the post-Gd T_1_-weighted MRI. The tumor volume for each patient at each scan time point is shown in Fig. S1 in supplementary material. The observed longitudinal relaxation rate, ${{\rm{R}}}_{{\rm{a}}}^{{\rm{obs}}}$, of each voxel was first calculated using the Method of Slopes^26,27^. Then, the two-pool MT model was fit to MRI data voxel-by-voxel for each patient at each time point and the four model parameters were calculated. Average parameter values for tumor and contralateral normal appearing white matter (cNAWM) regions were calculated and used in analyses. To illustrate the data and fitting performance, Fig. 1 shows the six MT spectrums as well as the fit to the data for a representative patient.

To minimize operator bias in selecting tumor and cNAWM boundaries, these ROIs were defined on the post-Gd T_1w_ image and then transferred to the qMT slice (by co-registering the two datasets). In order to assess the repeatability of the MT measurements and analysis, they were first performed on cNAWM (Table 1).

**Table 1:** Quantitative MT parameter values for cNAWM. Mean ± standard deviation across all subjects, calculated from average parameter value over cNAWM ROI, at each imaging time point.

Scan	$R$ $[{s}^{-1}]$	$R{M}_{0b}/{R}_{a}$	$1/({{\rm{R}}}_{{\rm{a}}}{{\rm{T}}}_{2{\rm{a}}})$	${T}_{2b}\,[\mu s]$	${R}_{{\rm{a}}}[{s}^{-1}]$	${M}_{0b}\,[ \% ]$
Day_0_	17.4 ± 2.3	2.89 ± 0.26	26.6 ± 4.5	11.4 ± 0.5	0.89 ± 0.07	15.0 ± 1.4
Day_14_	18.6 ± 3.3	2.81 ± 0.29	25.5 ± 4.9	11.5 ± 0.5	0.94 ± 0.07	14.6 ± 2.3
Day_28_	18.1 ± 2.8	2.93 ± 0.32	26.7 ± 4.6	11.3 ± 0.6	0.91 ± 0.07	15.0 ± 1.7
Day_70_	16.9 ± 1.6	2.80 ± 0.19	25.4 ± 2.8	11.4 ± 0.6	0.90 ± 0.07	15.1 ± 1.3

**Figure 2:**
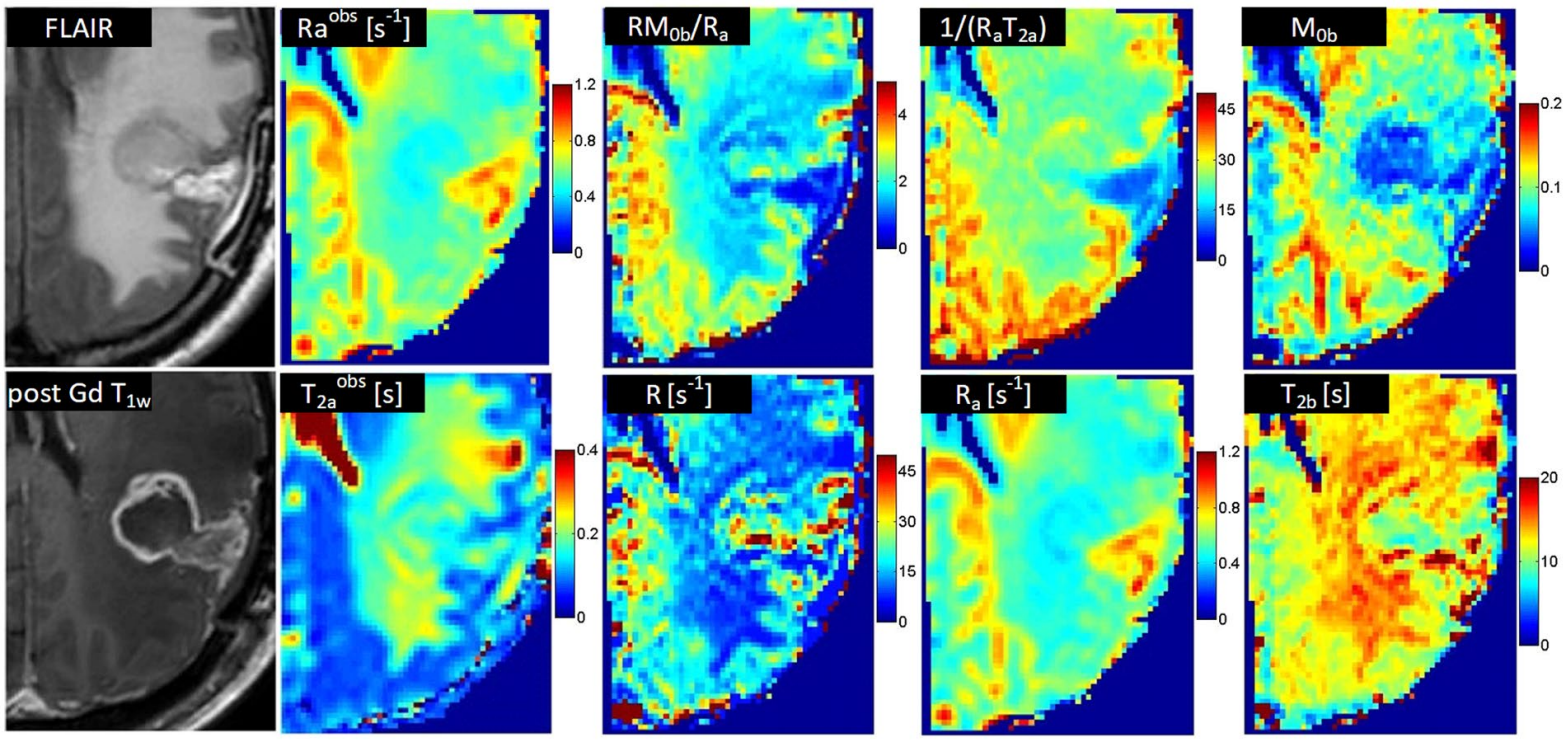
The FLAIR and post-Gd T_1w_ images of the tumor for a representative patient, as well as parametric maps of R_a_^obs^, T_2a_^obs^, and qMT parametric maps corresponding to RM_0b_/R_a_, R, 1/(R_a_T_2a_), M_0b_, and T_2b_.

There was no statistically significant difference between the MT model parameter of any two time-points or between progressors and non-progressors for cNAWM (assessed by unpaired t-test), demonstrating the stability and reproducibility of the experiments. Figure 2 shows the parametric maps for a representative patient. ${{\rm{R}}}_{{\rm{a}}}^{{\rm{obs}}}\,$and ${{\rm{T}}}_{2{\rm{a}}}^{{\rm{obs}}}$ are the observed ${{\rm{R}}}_{1}$ and ${{\rm{T}}}_{2}$ of the tissue and were measured independent of the qMT. The ${\rm{R}},\,{{\rm{T}}}_{2{\rm{b}}},\frac{{{\rm{RM}}}_{0{\rm{b}}}}{{{\rm{R}}}_{{\rm{a}}}},\frac{1}{{{\rm{R}}}_{{\rm{a}}}{{\rm{T}}}_{2{\rm{a}}}}$ are the four model parameters fitted by the qMT model, and ${{\rm{M}}}_{0{\rm{b}}}$ and ${{\rm{R}}}_{{\rm{a}}}$ were not fitted independently and were generated using the four qMT model parameters.

The histogram distribution of the two main qMT parameter representing both pools in the qMT model, i.e. amount of magnetization transfer (${{\rm{RM}}}_{0{\rm{b}}}/{{\rm{R}}}_{{\rm{a}}}$) and the direct effect of the free water pool ($1/({{\rm{R}}}_{{\rm{a}}}{{\rm{T}}}_{2{\rm{a}}})$) for each patient at baseline (Day_0_) and Day_14_ scans (for ROI type I) are shown in the supplementary material Figs S2 and S3. Several histogram-based metrics, i.e. mean, median, kurtosis, and skewness, were probed to represent the qMT parameter distribution in the selected ROI. Table S1 in supplementary material reports these histogram metrics for ${{\rm{RM}}}_{0{\rm{b}}}/{{\rm{R}}}_{{\rm{a}}}$ and $1/({{\rm{R}}}_{{\rm{a}}}{{\rm{T}}}_{2{\rm{a}}})$ in ROI type I and Table S2 in the supplementary material reports these parameter distributions for ROI type II. As can be seen in these tables and also in Figs S2 and S3, a Gaussian distribution is capable of representing the qMT parameter distribution in the ROI in majority of cases. Moreover, considering mean and median values are providing similar separation of the progressors and non-progressors shows that average parameter value over the ROI is an appropriate metric for the analysis.

Table 2 reports the distribution (mean and standard deviation) of model parameters for the tumor ROI type I in which tumor ROI was defined on enhancing region on the post-Gd T_1w_ image at each time point (segregated into progressors and non-progressors). The parameter pairs that were statistically significantly different between progressors and non-progressors are shown in bold.

**Table 2:** Quantitative MT parameter values for progressors (P) and non-progressors (NP). Mean ± standard deviation across all subjects, derived from average parameter value over tumor ROI defined at each imaging time point (ROI type I).

Scan		$R[{s}^{-1}]$	$R{M}_{0b}/{R}_{a}$	$1/({{\rm{R}}}_{{\rm{a}}}{{\rm{T}}}_{2{\rm{a}}})$	${T}_{2b}\,[\mu s]$	${R}_{{\rm{a}}}\,[{s}^{-1}]$	${M}_{0b}\,[ \% ]$
Day_0_	NP	22.5 ± 5.0	1.06 ± 0.24**	16.2 ± 5.5*	12.1 ± 1.2	0.79 ± 0.20	5.0 ± 0.5
	P	18.8 ± 3.5	**1.64 ± 0.48**	**24.3 ± 8.8**	11.6 ± 1.0	0.61 ± 0.15	6.6 ± 2.4
Day_14_	NP	19.3 ± 2.9	1.38 ± 0.39	22.7 ± 7.0	11.8 ± 1.1	0.63 ± 0.12	5.6 ± 1.6
	P	20.5 ± 2.3	1.77 ± 0.50	25.3 ± 5.1	12.1 ± 1.8	0.58 ± 0.07	6.6 ± 3.1
Day_28_	NP	19.8 ± 4.9	1.43 ± 0.26	22.2 ± 5.9	11.8 ± 1.2	0.61 ± 0.10	5.6 ± 1.2
	P	23.1 ± 8.4	1.71 ± 0.18	24.3 ± 3.4	11.0 ± 0.3	0.62 ± 0.17	5.8 ± 0.3
Day_70_	NP	22.0 ± 5.7	1.56 ± 0.40	24.1 ± 4.0	11.3 ± 1.5	0.56 ± 0.06	5.4 ± 1.4
	P	19.1 ± 7.4	1.68 ± 0.06	26.0 ± 3.6	10.3 ± 1.1	0.57 ± 0.14	6.0 ± 1.1

P: progressor, NP: non-progressor, *p < 0.05, **p < 0.01.

The values of ${{\rm{RM}}}_{0{\rm{b}}}/{{\rm{R}}}_{{\rm{a}}}$ (amount of magnetization transfer) at baseline (Day_0_) (p = 0.006), as well as $1/({{\rm{R}}}_{{\rm{a}}}{{\rm{T}}}_{2{\rm{a}}})$ (representing direct effect of free water pool) at baseline (p = 0.038) were statistically significantly different between progressors and non-progressors. There were no statistically significant differences between the two cohorts for any qMT parameter at any of the subsequent scans time-points. Table 3 reports the distribution (mean and standard deviation) of model parameters for the tumor ROI type II in which tumor ROI was defined on at Day_0_ scan and was kept the same for consecutive time points. For ROI type II, there were no statistically significant differences (similar to ROI type I) between progressors and non-progressors for any qMT parameters at any time point after the baseline scan.

**Table 3:** Quantitative MT parameter values for progressors (P) and non-progressors (NP). Mean ± standard deviation across all subjects, derived from average parameter value over tumor ROI defined at baseline and kept constant for subsequent time points (ROI type II).

Scan		$R[{s}^{-1}]$	$R{M}_{0b}/{R}_{a}$	$1/({{\rm{R}}}_{{\rm{a}}}{{\rm{T}}}_{2{\rm{a}}})$	${T}_{2b}\,[\mu s]$	${R}_{{\rm{a}}}\,[{s}^{-1}]$	${M}_{0b}\,[ \% ]$
Day_0_	NP	22.5 ± 5.0	1.06 ± 0.24**	16.2 ± 5.5*	12.1 ± 1.2	0.79 ± 0.20	5.0 ± 0.5
	P	18.8 ± 3.5	**1.64 ± 0.48**	**24.3 ± 8.8**	11.6 ± 1.0	0.61 ± 0.15	6.6 ± 2.4
Day_14_	NP	19.4 ± 3.0	1.55 ± 0.36	23.8 ± 7.0	11.5 ± 1.1	0.64 ± 0.10	6.4 ±1.8
	P	21.4 ± 2.9	1.93 ± 0.65	26.2 ± 4.7	11.7 ± 0.9	0.55 ± 0.04	6.8 ± 2.7
Day_28_	NP	20.3 ± 4.2	1.68 ± 0.27	23.0 ± 4.9	11.5 ± 0.8	0.62 ± 0.08	6.5 ± 1.7
	P	21.8 ± 7.6	1.85 ± 0.23	25.3 ± 2.7	11.2 ± 0.2	0.62 ± 0.15	6.8 ± 1.0
Day_70_	NP	21.2 ± 4.2	1.73 ± 0.35	23.7 ± 3.5	11.2 ± 0.8	0.60 ± 0.06	6.5 ± 1.3
	P	17.7 ± 3.7	1.70 ± 0.11	25.8 ± 2.3	11.0 ± 0.6	0.56 ± 0.09	6.5 ± 1.0

P: progressor, NP: non-progressor, *p < 0.05, **p < 0.01.

**Figure 3:**
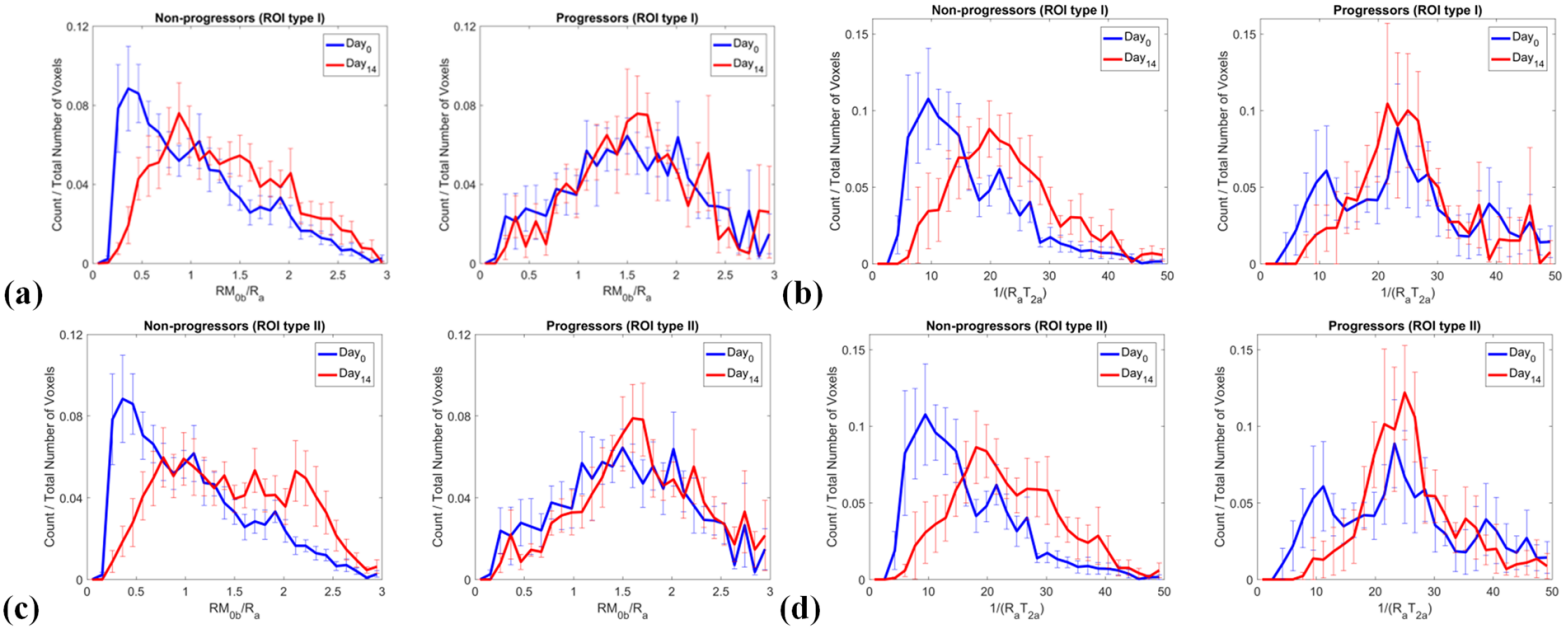
Summary histogram plots of qMT parameters for all patients in each cohort for (**a,b)** ROI type I and (**c,d)** ROI type II showing that between Day_14_ and Day_0_ the parameters increased for non-progressors but were relatively unchanged for progressors. Comparing the Day_14_ plots of amount of magnetization transfer (RM_0b_/R_a_) for non-progressors between ROI type II and ROI type I shows that, there is an increase in the larger value portion (RM_0b_/R_a_ > 1.5) for ROI type II, which demonstrates the white matter entering the tumor area in non-progressors (RM_0b_/R_a_ has larger values for white matter compared to the tumor). The plots represent the mean and standard error of histogram probabilities of individual patients (histogram count normalized with respect to the number of voxels in the ROI) which are shown in supplementary material Figs S2 and S3.

The summary of individual parameter histograms (Figs S2 and S3) corresponding to ${{\rm{RM}}}_{0{\rm{b}}}/{{\rm{R}}}_{{\rm{a}}}$ and $1/({{\rm{R}}}_{{\rm{a}}}{{\rm{T}}}_{2{\rm{a}}})$ for each cohort at Day_0_ and Day_14_ were combined by plotting the mean and standard error of these histogram distributions for each histogram bin value (normalized with respect to the number of voxels in the ROI). Figure 3a,b show the summary histogram plot for progressors and non-progressors in ROI type I, showing that there was an increase in parameter values for progressors while for non-progressors these qMT parameters were unchanged between Day_0_ and Day_14_. Similar plots and trends are shown in Fig. 3c,d for ROI type II.

**Figure 4:**
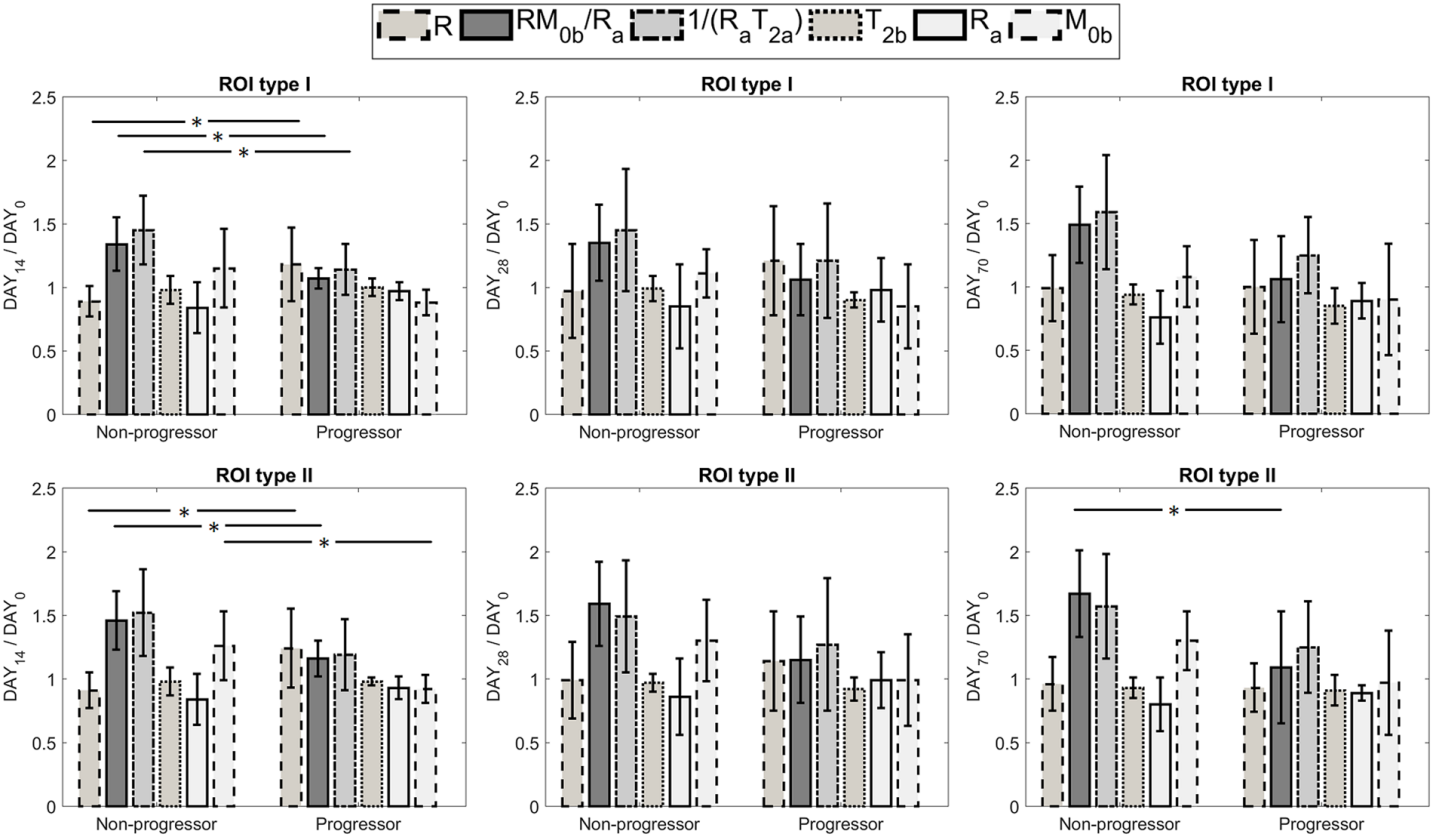
The ratio of each parameter value at Day_14_, Day_28_ and Day_70_ over its value at baseline (Day_0_) for both ROI type I and ROI type II, segregated for progressors and non-progressors. The parameters with statistically significantly different between the two cohorts are identified (*p < 0.5, **p < 0.01).

Figure 4 shows the distribution of ratio of each parameter (at Day_14_, Day_28_ and Day_70_) over its value at baseline (Day_0_) for progressors and non-progressors. This figure represents the change in each parameter due to treatment. For ROI type I, the parameter ratios that were statistically significantly different between progressors and non-progressors were the amount of magnetization transfer at Day_14_ (relative ${{\rm{RM}}}_{0{\rm{b}}}/{{\rm{R}}}_{{\rm{a}},{\rm{Non}}-{\rm{progressor}}}$ = 1.34 ± 0.21, relative ${{\rm{RM}}}_{0{\rm{b}}}/{{\rm{R}}}_{{\rm{a}},{\rm{Progressor}}}$ = 1.07 ± 0.08, p = 0.031), the exchange rate at Day_14_ (relative ${{\rm{R}}}_{,{\rm{Non}}-{\rm{progressor}}}$ = 1.18 ± 0.29, relative ${{\rm{R}}}_{,{\rm{Progressor}}}$ = 0.89 ± 0.12, p = 0.025), and the direct effect of the free water pool at Day_14_ (relative $1/{({{\rm{R}}}_{{\rm{a}}}{{\rm{T}}}_{2{\rm{a}}})}_{,{\rm{Non}}-{\rm{progressor}}}$ = 1.45 ± 0.27, relative $1/{({{\rm{R}}}_{{\rm{a}}}{{\rm{T}}}_{2{\rm{a}}})}_{,{\rm{Progressor}}}$ = 1.14 ± 0.20, p = 0.049).

For ROI type II, the parameter ratios that were statistically significantly different between progressors and non-progressors were: the amount of magnetization transfer at Day_14_ (relative ${{\rm{RM}}}_{0{\rm{b}}}/{{\rm{R}}}_{{\rm{a}},{\rm{Non}}-{\rm{progressor}}}$ = 1.46 ± 0.23, relative ${{\rm{RM}}}_{0{\rm{b}}}/{{\rm{R}}}_{{\rm{a}},{\rm{Progressor}}}$ = 1.16 ± 0.14, p = 0.033), the size of the macromolecular pool at Day_14_ (relative ${{\rm{M}}}_{0{\rm{b}},{\rm{Non}}-{\rm{progressor}}}$ = 1.26 ± 0.27, relative ${{\rm{M}}}_{0{\rm{b}},{\rm{Progressor}}}$ = 0.92 ± 0.11, p = 0.040), and the exchange rate at Day_14_ (relative ${{\rm{R}}}_{,{\rm{Non}}-{\rm{progressor}}}$ = 0.91 ± 0.14, relative ${{\rm{R}}}_{,{\rm{Progressor}}}$ = 1.24 ± 0.31, p = 0.020) as well as the amount of magnetization transfer at Day_70_ (relative ${{\rm{RM}}}_{0{\rm{b}}}/{{\rm{R}}}_{{\rm{a}},{\rm{Non}}-{\rm{progressor}}}$ = 1.67 ± 0.34, relative ${{\rm{RM}}}_{0{\rm{b}}}/{{\rm{R}}}_{{\rm{a}},{\rm{progressor}}}$ = 1.09 ± 0.44, p = 0.031).

**Figure 5:**
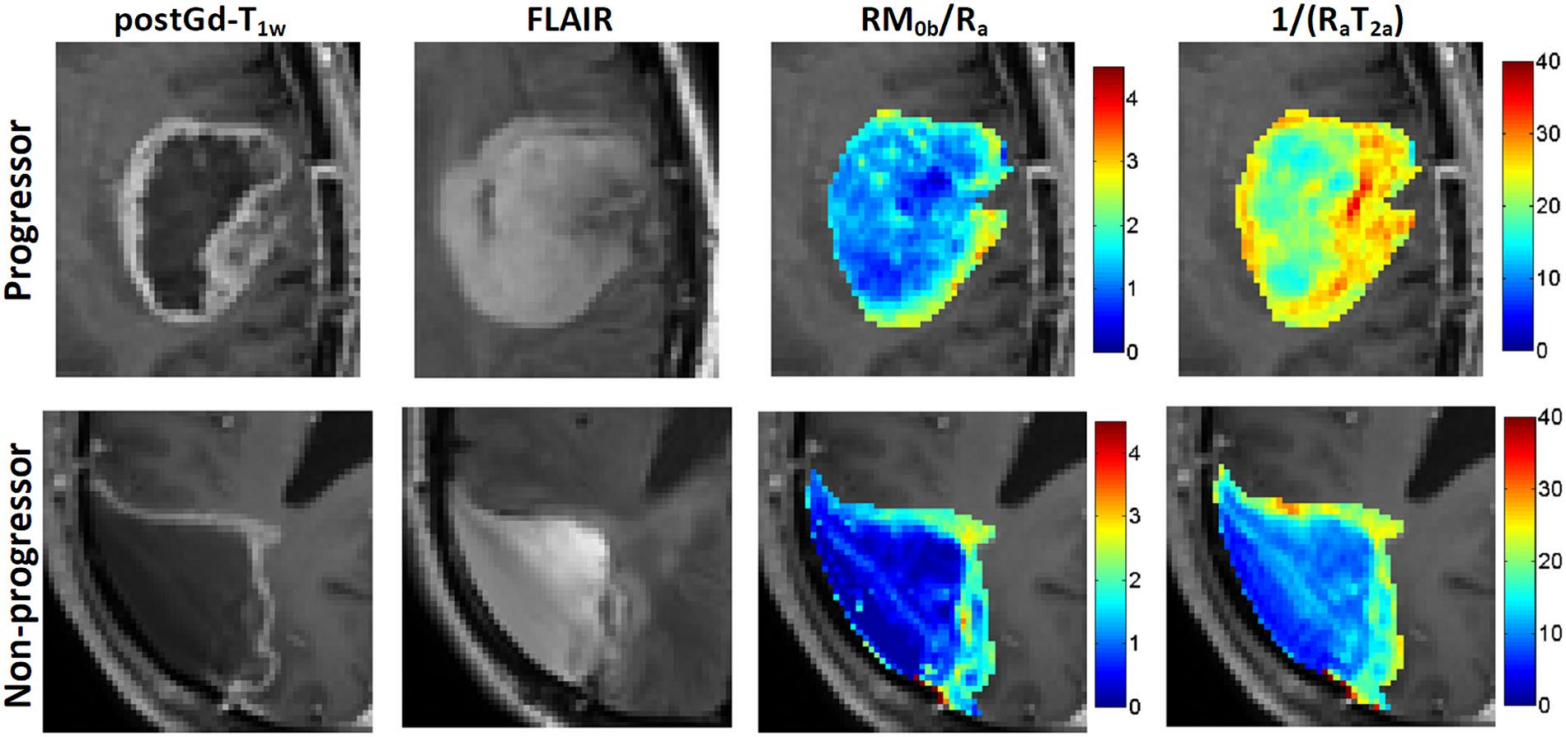
The parametric maps of RM_0b_/R_a_ and 1/(R_a_T_2a_) overlaid on the post-Gd T_1w_ images of a representative patient with non-progressive tumor and a representative patient with progressive tumor.

**Figure 6:**
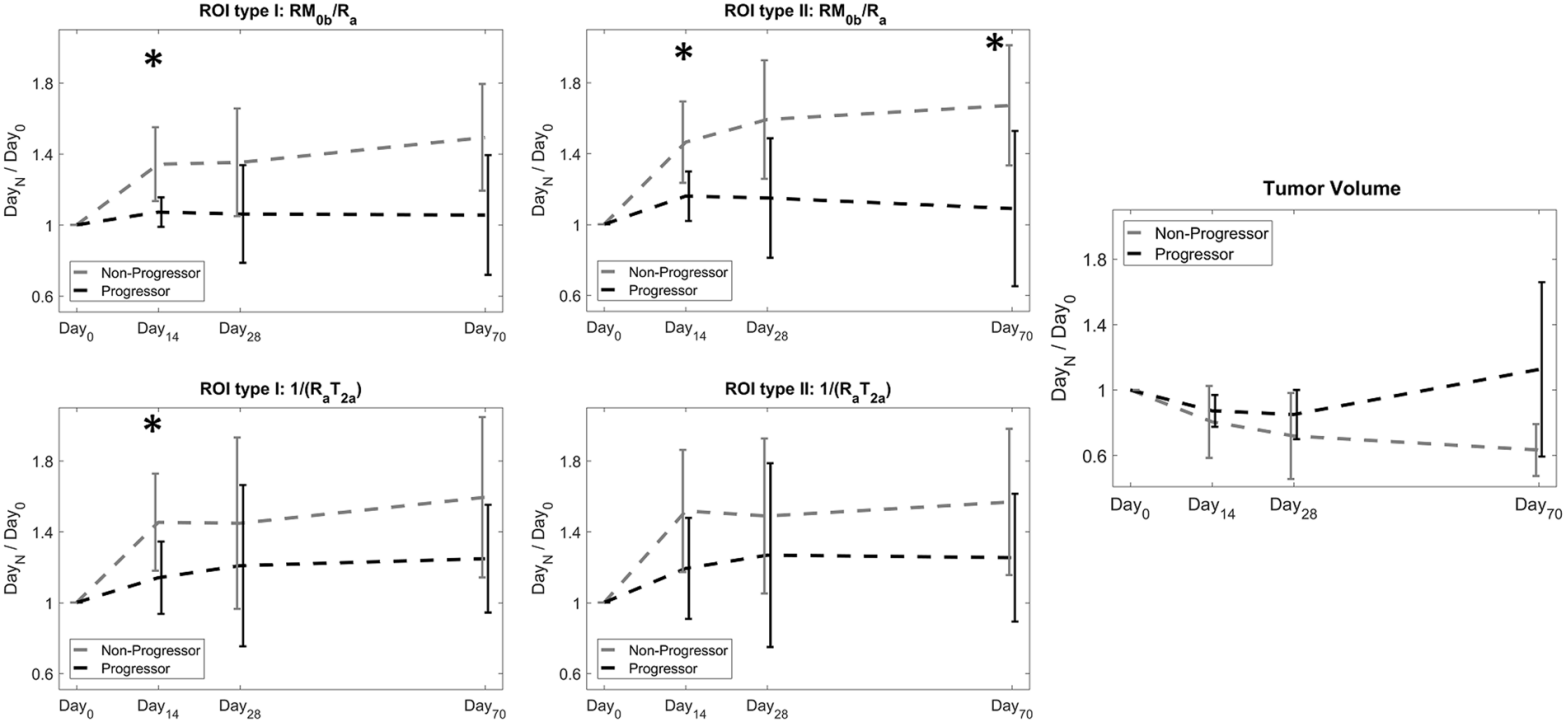
The mean and standard deviation for normalized RM_0b_/R_a_ and normalized 1/(R_a_T_2a_) that were capable of separating the progressors and non-progressors as well as the normalized tumor volume. These plots show the ratio of the parameter value at each time point over its value at baseline (Day_0_).

Figure 5 shows the parametric maps of ${{\rm{RM}}}_{0{\rm{b}}}/{{\rm{R}}}_{{\rm{a}}}$ and $1/({{\rm{R}}}_{{\rm{a}}}{{\rm{T}}}_{2{\rm{a}}})$ - whose normalized values provided the best separation of progressors from non-progressors (Fig. 4) - overlaid on post-Gd T_1w_ images corresponding to the Day_0_ scan for a progressor and a non-progressor. This figure shows that the progressor and non-progressor have different spatial distributions of these parameters. For the non-progresor case, high MT (${{\rm{RM}}}_{0{\rm{b}}}/{{\rm{R}}}_{{\rm{a}}}$) was measured at the tumor rim with very little MT in the tumor core, while for the progressor, high MT was measured in both rim and core of the tumor. Figure 6 plots the change (over time) in the two parameters ratios, ${{\rm{RM}}}_{0{\rm{b}}}/{{\rm{R}}}_{{\rm{a}}}$ and $1/({{\rm{R}}}_{{\rm{a}}}{{\rm{T}}}_{2{\rm{a}}})$, that were statistically significantly different between progressor and non-progressors.

## Discussion

Therapeutic response of human GBM to chemo-radiation was studied longitudinally. Nineteen patients with newly diagnosed GBM were recruited and repeatedly MRI-scanning over the course of their 6-week treatment (2 Gy/day radiotherapy concurrent with daily chemotherapy with Temozolomide). The objectives were to:

- Characterize the MT properties of the GBM tumor and their evolution over the course of the treatment.
- Determine the **earliest** time point at which qMT parameters were able to identify early tumor progression.
- Identify the qMT parameters that were able to characterize tumor aggressiveness **before** the start of the treatment.

For each scan of each patient, an ROI in the cNAWM was selected and MT modeling was performed. The MT model parameters for cNAWM at each time point were reported in Table 1, demonstrating there were no statistically significant differences between the values at different time points (no intra-subject variation). Also, the small variations in the parameters of each time point showed that the values were similar between patients (no inter-subject variation). These results warrant the repeatability of the experiments and that the differences that were observed between tumors were due to changes in tumors and not differences in experimental conditions.

The analysis ROI was defined on the post-Gd T_1w_ MRI. qMT parameters of the tumor were investigated in two ROI types to provide a comprehensive assessment of the treatment-induced effects. ROI type I focused on the tumor tissue only and reflected the qMT parameters inside the enhancing rim of the tumor over time. ROI type II was defined at Day_0_ scan (which was used for radiation treatment planning) and was kept constant for the subsequent time points. Thus, ROI type II received the highest radiation dose throughout the 6-week treatment. qMT characteristics of this ROI reflected the changes in tumor size and also the white matter infiltration into the initial tumor area during the treatment.

As reported in Tables 1, 2 and 3, the amount of magnetization transfer of the GBM tumor was statistically significantly smaller than the cNAWM (${{\rm{RM}}}_{0{\rm{b}}}/{{\rm{R}}}_{{\rm{a}},{\rm{GBM}}}$ = 1.28 ± 0.44, ${{\rm{RM}}}_{0{\rm{b}}}/{{\rm{R}}}_{{\rm{a}},{\rm{cNAWM}}}$ = 2.89 ± 0.26, p < 0.001) which is consistent with previous studies^20,22,25^. This difference resulted from smaller macromolecular pool in tumors (resulting from the smaller lipid content of the tumor compared to cNAWM) compared to cNAWM (${{\rm{M}}}_{0{\rm{b}},{\rm{GBM}}}$ [%] = 5.6 ± 1.6, ${{\rm{M}}}_{0{\rm{b}},{\rm{cNAWM}}}$ [%] = 15.0 ± 1.4, p < 0.001). The difference of ${{\rm{M}}}_{0{\rm{b}}}$ was so large that it counteracted the higher intrinsic relaxation rate, ${{\rm{R}}}_{{\rm{a}}}$ (${{\rm{R}}}_{{\rm{a}},{\rm{GBM}}}$ [s^−1^] = 0.72 ± 0.20, ${{\rm{R}}}_{{\rm{a}},{\rm{cNAWM}}}$ [s^−1^] = 0.89 ± 0.07, p = 0.008), and lower exchange rate, R (${{\rm{R}}}_{,{\rm{GBM}}}$ [s^−1^] = 21.1 ± 4.7, ${{\rm{R}}}_{,{\rm{cNAWM}}}$ [s^−1^] = 17.4 ± 2.3, p = 0.015) in cNAWM compared to tumor.

Although the general trends in qMT parameters in Tozer *et al*.^20^ were similar to the current study, the absolute parameter values, in particular, 1/(R_a_T_2a_), were different (even for white matter). This could be due to the fact that Tozer applied the closed-form solution for two-pool MT model presented by Henkelman *et al*.^28^, which is for steady-state saturation MT, to calculate the qMT parameters. However, the SAR and hardware limitations in human scanners do not allow long enough saturation to reach steady-state. To overcome this issue, we calculated the qMT parameters in transient state through solving the bloch-McConnel equations for the two-pool MT model. As a result, our parameter values for white matter were similar to those reported previously by Morrison *et al*.^29^ and Levesque *et al*.^30^.

As reported in Tables 2 and 3, there was a statistically significant difference in the amount of magnetization transfer between the progressors and non-progressors at baseline (Day_0_) before the start of the treatment (${{\rm{RM}}}_{0{\rm{b}}}/{{\rm{R}}}_{{\rm{a}},{\rm{Non}}-{\rm{progressor}}}$ = 1.06 ± 0.24, ${{\rm{RM}}}_{0{\rm{b}}}/{{\rm{R}}}_{{\rm{a}},{\rm{Progressor}}}$ = 1.64 ± 0.48, p = 0.006). Non-progressors had a lower amount of MT at baseline and treatment resulted in a large increase in this parameter. However, for progressors, the amount of MT was higher and the treatment was not able to change it significantly (Figs 4 and 6). Moreover, direct effect of free water pool was lower in non-progressors ($1/{({{\rm{R}}}_{{\rm{a}}}{{\rm{T}}}_{2{\rm{a}}})}_{,{\rm{Non}}-{\rm{progressor}}}$ = 16.2 ± 5.5) compared to progressors ($1/{({{\rm{R}}}_{{\rm{a}}}{{\rm{T}}}_{2{\rm{a}}})}_{,{\rm{Progressor}}}$ = 24.3 ± 8.8) with p = 0.038. Considering ${{\rm{R}}}_{{\rm{a}}}$ was not different between the two cohorts, transvers relaxation of the free water pool, ${{\rm{T}}}_{2{\rm{a}}}$, was higher in non-progressors, suggesting they had higher water content and lower cellular density. These results demonstrate the ability of qMT in determining GBM tumors that are resistant to standard chemo-radiation treatment even before the start of the treatment.

The ratio of each qMT parameter at each time point over its baseline (Day_0_) value represented the treatment-induced changes in the tumor. As shown in Figs 4 and 6, the change in ${{\rm{RM}}}_{0{\rm{b}}}/{{\rm{R}}}_{{\rm{a}}}$ and $1/({{\rm{R}}}_{{\rm{a}}}{{\rm{T}}}_{2{\rm{a}}})$ (between baseline and Day_14_ scans) were statistically significantly different between progressors and non-progressors for ROI type I with p = 0.025 and p = 0.049 respectively. These two parameters reflect the qMT properties of both proton pools in the model (${{\rm{RM}}}_{0{\rm{b}}}/{{\rm{R}}}_{{\rm{a}}}$ for the bound macromolecular pool and $1/({{\rm{R}}}_{{\rm{a}}}{{\rm{T}}}_{2{\rm{a}}})$ for the free water pool), showing that the GBM treatment is affecting both pools. When considering ROI type II which represented the region that received highest radiation dose throughout treatment, the ratio of most of the parameters of the macromolecular pool at Day_14_ over Day_0_ were statistically significantly different between the two cohorts (${{\rm{RM}}}_{0{\rm{b}}}/{{\rm{R}}}_{{\rm{a}}}$, ${{\rm{M}}}_{0{\rm{b}}}$, and ${\rm{R}}$ with p = 0.033, p = 0.040, and p = 0.040 respectively).

Interestingly, beyond the two-week time point, these parameters remained relatively constant or got closer to each other (weakening the separation). Thus, the best time point to evaluate GBM response to treatment was within two weeks into the treatment, and the MT parameters measured at later time points were unable to predict response. This also demonstrated that qMT parameters were much more sensitive to treatment and changed significantly at early days of the treatment.

The difference between qMT parameters of the progressors and non-progressors was even more pronounced when considering their changes in the initial tumor boundaries (ROI type II) as can be seen in Figs 4 and 6. These figures show that the qMT parameters of non-progressors increased, while for progressors they remained relatively unchanged, and at day_70_ they had slightly lower values than baseline. We hypothesize that the increased qMT parameter values for non-progressor was due to tumor response resulting in a greater proportion of white matter contributing to their value (due to tumor shrinkage or white matter entering the tumor ROI). This point demonstrates that (**a)** tumor boundaries in post-Gd T_1w_ MRI do not properly reflect that extent of tumor and, (**b)** tumor aggressiveness can be better characterized when considering the initial tumor boundaries. Moreover, the tumor volume was not able to separate progressors from non-progressors at any of the time-points highlighting the need for longer follow-up when using biomarkers that are based on tumor size.

The main limitation of this study was its small sample size. Although the differences between qMT properties of the two cohorts were large, there were only six patients with progressive tumors at baseline and only four participated in the follow-up scans. A larger number of progressors are needed to increase confidence in the results. Another major challenge was the long scan time and the fact that a single slice through the tumor was investigated. The imaging slice was selected such that it covered the largest cross section of the tumor, covering 1.1 [cm^3^] to 5.9 [cm^3^] of the tumor volume which represented 8% to 21% of the total tumor volume of the patient. We acquired six MT spectrums to characterize the MT parameters accurately, however, MT quantification could be performed with fewer spectrums, which would enable imaging more than one slice in the tumor and result in better quantification of the disease. A subsequent larger study is in progress to confirm these results.

These findings have the potential for significant clinical utility in the era of MRI-based image-guided radiotherapy. As we image patients daily prior to each radiotherapy session, if the MT does not change, then this may be a biomarker to dose-escalate or change the systemic therapy adjuvantly. There are several possibilities to personalize treatment options with a reliable biomarker of response and qMT requires further evaluation to confirm our result. It is important to note the lack of contrast required for this acquisition is a major advantage as with daily MR imaging the patient cannot be administered contrast regularly.

## Materials and Methods

### Study Design

Informed consent was obtained from all patients under an institutional research ethics board (REB) approved protocol. The study was conducted in accordance with regulations and guidelines of REB and all experimental protocols were approved by the REB at Sunnybrook health sciences centre, Toronto, Canada. Nineteen patients with newly diagnosed GBM were recruited (13 were males and the median age was 55 years). All patients underwent concurrent radiation with 60 Gy in 30 fractions (2 Gy/day) concurrent with daily Temozolomide over six weeks. Isocitrate dehydrogenase 1 (IDH1) gene mutation status was determined for 17 out of 19 patients, where IDH1 mutation was not determined for one progressor and one non-progressor, two of the non-progressors had mutant IDH1 gene, and the other 15 had wild-type IDH1. O^6^-methylguanine-DNA methyltransferase (MGMT) promoter methylation status was not determined for the patients as a standard test at our institution.

Each patient was MRI scanned at four time points: (1) immediately before the start of the treatment (Day_0_), (2) After receiving 10 treatment sessions (Day_14_), (3) After receiving 20 treatment sessions (Day_28_), and (4) four weeks after the end of the treatment (Day_70_).

Patients were grouped into early progressors or non-progressors by a senior neuro-oncologist that was blinded to the quantitative MRI analysis. Progression status was determined more than 3 months (3 to 8 months) after the end of the chemo-radiation treatment, and was defined as per the RANO criteria by assessing the changes in tumor size on post-Gadolinium (Gd) T_1_-weighted and T_2_-weighted FLAIR MRI, as well as clinical symptoms of the patient^7^. In order to avoid misclassifying patients with pseudo-progression as progressors, RANO criteria was used which is specifically designed to address the issue of pseudoprogression through considering strict criteria for determining progression within the first 12 weeks of the treatment^7^.

### MRI Acquisition

The patients were scanned on a 3T Philips Achieva MRI system with 8-channel SENSE head coil with the following MRI sequences:

The first acquired sequence was T_2_-weighted FLAIR which involved 25 slices with 5 mm thickness and field of view (FOV) of 24 cm × 2 4 cm to cover the entire brain (TR/TE/TI = 9000/2800/125 ms).

Using the FLAIR images, an oblique axial slice passing through the largest cross-section of the tumor was chosen for MT imaging. MT spectrum images were acquired for fourteen offset frequencies. The first two images in the spectrum were acquired at 100 kHz (780 ppm) offset and were averaged to generate the reference image. The remaining twelve offset frequencies covered the range between 100 kHz and 250 Hz (~2 ppm) with logarithmic spacing (63 kHz, 40 kHz, 25 kHz, 16 kHz, 10 kHz, 6.3 kHz, 4.0 kHz, 2.5 kHz, 1.0 kHz, 0.63 kHz, 0.4 kHz, 0.25 kHz). The MT spectrum images were acquired with six unique combinations of RF power amplitudes (${B}_{1}$ = 1.5/3.0/5.0 µT) and saturation durations (${T}_{{\rm{sat}}}$ = 485/970 ms). The RF saturation consisted of two or four block-shaped pulses of 242.5 ms each. There was also a delay of 2.5 ms after each block, during which spoilers were applied in the slice selection direction.

A fast field echo (FFE) sequence was used with multi-shot Turbo Field Echo (TFE), TFE factor = 20, TR/TE = 7.78/4.5 ms, half scan = 0.8, Acquisition Matrix = 132 × 95, Reconstruction Matrix = 144 × 144, FOV = 20 cm × 20 cm, slice thickness = 3 mm. There was also a SPIR fat suppression (12 ms) after the saturation pulses and before the TFE acquisition. In order to allow for the magnetization to recover and also to satisfy duty cycle constraints, a delay was included after TFE acquisition, making the time between consecutive saturations equal to 1000 ms and 2000 ms, for RF ${T}_{{\rm{sat}}}$ of 485 ms and 970 ms respectively. The time between consecutive images for MT spectrums with ${B}_{1}$ = 5.0 µT were longer due to hardware and specific absorption rate (SAR) limitations (4481 ms for ${T}_{{\rm{sat}}}$ = 970  ms, and 2000 ms for ${T}_{{\rm{sat}}}$ = 485 ms). The total duration of MT acquisition (for the six spectrums) was 3.1 min.

${T}_{2}$-mapping was performed on the same slice using a ${T}_{2}$-weighted spin echo sequence with 10 echo times (TE = n × 20 ms, n = 1, 2, …, 10), TR = 3000 ms, FOV = 20 cm × 20 cm, slice thickness = 3 mm, matrix size = 80 × 80, ${\rm{\alpha }}$ = 90°. ${T}_{2}$-mapping was performed by fitting a mono-exponential function to the data on a voxel-by-voxel basis.

The method of Slopes (MoS) was used for ${{\rm{B}}}_{1}$- and ${R}_{1}$-mapping^26^. MoS image acquisition consisted of high spatial resolution images with small flip angles (FFE, ${\rm{\alpha }}$ = 3°, 14°, TR/TE = 10.7 ms/5 ms, FOV = 20 cm × 20 cm, matrix size = 224 × 224 × 40, Slice Thickness = 2 mm), as well as low spatial resolution images with large flip angles (FFE, ${\rm{\alpha }}$ = 130°, 150°, TR/TE = 50 ms/5 ms, FOV = 20 cm × 20 cm, matrix size = 80 × 80 × 20, Slice Thickness = 6 mm). The low resolution, high flip angle images were used for ${{\rm{B}}}_{1}$-mapping and the high resolution, low flip angle data allowed for high resolution ${R}_{1}$-mapping^27,31^.

For the last scan, a bolus of contrast agent (gadobutrol, Bayer Inc., Toronto, Canada) was injected intravenously at a dose of 0.1 mmol/kg of patient’s body weight followed by a 20 mL saline flush. Then, high spatial resolution post-contrast 3D Axial ${{\rm{T}}}_{1}$-weighted imaging was performed with the following sequence parameters: TR/TE = 9.5 ms/2.3 ms, ${\rm{\alpha }}$ = 8°, FOV = 22 cm × 22 cm, matrix size = 448 × 448 × 113, slice thickness = 1.5 mm. This sequence was used for clinical assessment of tumor volume as well as delineating the lesion region of interest (ROI) for the MT analysis.

### MT modeling

A two-pool tissue model consisting of the free water pool and the semi-solid macromolecular pools was used^28,29^. The closed-form signal equation presented by Henkelman^28^ assumes that the MT-prepared magnetization has reached steady-state. However, it is not practical to satisfy this condition using clinical scanners in patients (due to SAR and hardware limitations). The qMT modeling was performed in transient state using the Bloch-McConnel equations as follows^32–34^:

1 $$\{\begin{array}{c}\begin{array}{c}\frac{d{M}_{Xa}}{dt}=-\frac{{M}_{Xa}}{{T}_{2a}}-2\pi {\rm{\Delta }}{M}_{Ya}\,\\ \frac{d{M}_{Ya}}{dt}=-\frac{{M}_{Ya}}{{T}_{2a}}+2\pi {\rm{\Delta }}{M}_{Xa}-{\omega }_{1}{M}_{Za}\,\\ \frac{d{M}_{Za}}{dt}={R}_{a}({M}_{0a}-{M}_{Za})-R{M}_{0b}{M}_{Za}+R{M}_{0a}{M}_{Zb}+{\omega }_{1}{M}_{Ya}\,\end{array}\\ \frac{d{M}_{Zb}}{dt}={R}_{b}({M}_{0b}-{M}_{Zb})-R{M}_{0a}{M}_{Zb}+R{M}_{0b}{M}_{Za}-{R}_{rfb}{M}_{Zb}\end{array}$$

where ${\rm{\Delta }}$ is the frequency offset and ${\omega }_{1}\,$is the amplitude of the saturation pulse. $R$ is the MT exchange rate constant between the two pools, ${R}_{a}$ and ${R}_{b}$ are the longitudinal relaxation rates, and ${M}_{0a}$ and ${M}_{0b}\,$are proportional to spin population in each pool. ${M}_{Xa},{M}_{Ya},{M}_{Za}$ are the longitudinal and transverse magnetization terms of the water pool, and magnetization in semisolid pool is approximated by the longitudinal component^35,36^. MT exchange effects on the semi-solid pool are expressed with the absorption rate constant ${{\rm{R}}}_{{\rm{rfb}}}$^37^ for which we used a super Lorentzian line-shape given by:

2 $${{\rm{R}}}_{{\rm{rfb}}}={{\rm{\pi }}{\rm{\omega }}}_{1}{\int }_{0}^{{\rm{\pi }}/2}\,\sin \,{\rm{\theta }}\sqrt{\frac{2}{{\rm{\pi }}}}\frac{{{\rm{T}}}_{2{\rm{b}}}}{|3\,{\cos }^{2}\,{\rm{\theta }}-1|}\exp [-2{(\frac{2{{\rm{\pi }}T}_{2{\rm{b}}}}{|3{\cos }^{2}{\rm{\theta }}-1|})}^{2}]d{\rm{\theta }}$$

As pointed out by Henkelman *et al*.^28^ in order to determine the model parameters, ${{\rm{R}}}_{{\rm{a}}}$, the longitudinal relaxation of the free water pool (without interference of the semi-solid pool), has to be determined independently. This relaxation rate was determined by measuring ${{\rm{R}}}_{{\rm{a}}}^{{\rm{obs}}}$, the observed longitudinal relaxation rate of the combined two-pool system (which includes the effects of exchange and semi-solid pool) and using eq. [3]^28^:

3 $${{\rm{R}}}_{{\rm{a}}}=\frac{{{\rm{R}}}_{{\rm{a}}}^{{\rm{obs}}}}{1+(\frac{[\frac{{{\rm{RM}}}_{0{\rm{b}}}}{{{\rm{R}}}_{{\rm{a}}}}]({{\rm{R}}}_{{\rm{b}}}-{{\rm{R}}}_{{\rm{a}}}^{{\rm{obs}}})}{({{\rm{R}}}_{{\rm{b}}}-{{\rm{R}}}_{{\rm{a}}}^{{\rm{obs}}})+{\rm{R}}})}$$

${{\rm{R}}}_{{\rm{a}}}^{{\rm{obs}}}$ for each voxel was measured through ${{\rm{R}}}_{1}$-mapping with MoS. The longitudinal relaxation rate of the semi-solid pool, ${{\rm{R}}}_{{\rm{b}}}$, was fixed to unity^28,29,38^, and therefore the system could be represented with four independent parameters $[{\rm{R}},{{\rm{T}}}_{2{\rm{b}}},\frac{{{\rm{RM}}}_{0{\rm{b}}}}{{{\rm{R}}}_{{\rm{a}}}},\frac{1}{{{\rm{R}}}_{{\rm{a}}}{{\rm{T}}}_{2{\rm{a}}}}]$. The differential equations in eq. [1] were fit voxel-by-voxel to the data in transient state using matrix exponentials and through Levenberg-Marquardt algorithm.

### Data Analysis

MT images of all six MT spectrums, the multi echo images for ${T}_{2}$-mapping, and the SPGR images for ${R}_{1}$/${B}_{1}$-mapping were all co-registered to the first acquired image (first reference image corresponding to ${B}_{1}=5\,{\rm{\mu }}T$) using affine registration in Elastix^39^. The images of each saturation power were them normalized with respect to their reference image and then fit to the MT model.

### Tumor ROI

The 3D images of the post-Gd T_1__w_ MRI were first co-registered to the 3D FLAIR images using affine registration in Elastix. They were then interpolated with the voxel resolution of the MT data and the oblique axial slice corresponding to MT was reconstructed. Analysis ROIs were then defined on this post-Gd T_1w_ slice. Two ROI types were used in longitudinal evaluation of the qMT parameters of the tumor:

- ROI type I: The tumor ROI was defined as the enhancing region on the post-Gd T_1w_ slice that was acquired at each scan
- ROI type II: The tumor ROI was defined as the enhancing region on the post-Gd T_1w_ slice at the baseline scan and was kept constant for the subsequent scans.

ROI type I evaluated the qMT parameters on the tumor tissue and did not take into account the changes in the tumor size. ROI type II on the other hand took the change in tumor size into account. By investigating both ROI type we obtained a comprehensive understanding of the qMT parameter changes over the course of the treatment.

### Normal tissue

The qMT parameters were also calculated on an ROI of normal appearing white matter on the contralateral side of the brain (cNAWM). This analysis was performed to assess the reproducibility of the qMT parameters between patients and also over the course of the treatment (inter-subject and intra-subject reproducibility). An ROI with uniform signal intensity in the white matter was selected on the post-Gd T_1w_ slice as the cNAWM.

The average qMT parameter value was calculated for each ROI and was used for in subsequent statistical analyses. Statistical significance of the differences between parameter distributions were performed using a two-sample t-test. This unpaired test was selected as the number of cases in the two cohorts were not equal (the significance level was set at p < 0.05).

### Histogram Analysis

The distribution of qMT parameters in the ROIs might not be Gaussian which necessitates performing histogram analysis. The histogram of the two main qMT parameter distributions, i.e. amount of magnetization transfer (${{\rm{RM}}}_{0{\rm{b}}}/{{\rm{R}}}_{{\rm{a}}}$) and the direct effect of the free water pool ($1/({{\rm{R}}}_{{\rm{a}}}{{\rm{T}}}_{2{\rm{a}}})$) were studied. Considering there were different numbers of voxels in different ROIs, each histogram bin count was divided by the total number of voxels in the ROI (providing the probability distribution of the parameter value and thus removing the effect of different tumor sizes in the histogram bin sizes).

Summary histogram plot of the histograms of individual scans were provided by calculating the mean and standard error of histogram probabilities for each parameter value (segregated for progressors and non-progressors at each scan) and plotting this average qMT parameter distribution. These plots demonstrated how each parameter was changing between different scans for each cohort.

### Ethical Approval and Informed Consent

The study was approved by the research ethics board of the Sunnybrook Health Sciences center. All patients provided informed consent to participate in the study.

### Data Availability Statement

Data were collected and available at the Odette Cancer Centre, Sunnybrook Health Sciences Centre, Toronto, Ontario, Canada.

## Electronic supplementary material

Supplementary Information
