# Supplementary material for: Quantitative Magnetization Transfer in Monitoring Glioblastoma (GBM) Response to Therapy
Source: Sci Rep. 2018 Feb 6;8:2475. doi: 10.1038/s41598-018-20624-6 (PMC5802834; doi:10.1038/s41598-018-20624-6)
Supplement: Supplementary file 1 — Supplementary Information [file 41598_2018_20624_MOESM1_ESM.pdf]

Supplementary Material

Quantitative Magnetization Transfer in Monitoring Glioblastoma (GBM) Response to Therapy

Hatef Mehrabian *[1,2], Sten Myrehaug[3,4], Hany Soliman[3,4], Arjun Sahgal[2,3,4], Greg J. Stanisz[1,2,5]

[1]Medical Biophysics, University of Toronto, Toronto, Ontario, Canada,

[2]Physical Sciences, Sunnybrook Research Institute, Toronto, Ontario, Canada

[3]Radiation Oncology, Sunnybrook Health Sciences Centre, Toronto, Ontario, Canada

[4]Department of Radiation Oncology, University of Toronto, Toronto, Ontario, Canada

[5]Department of Neurosurgery and Pediatric Neurosurgery, Medical University, Lublin, Poland

* hatef.mehrabian@sunnybrook.ca

Supplementary Figures and Tables

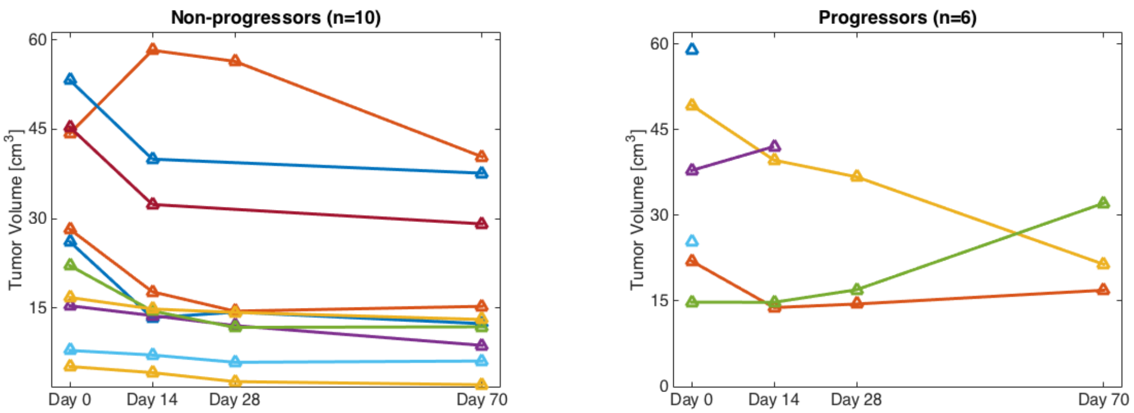

(a) (b)

Figure S-1 Tumor volume measured from enhancing region in post-Gd T_1-weighted MRI, shown for each time point of each patient. Each color represents one patient and symbols represent the actual volume measurements (two of the progressors participated in the first scan only).

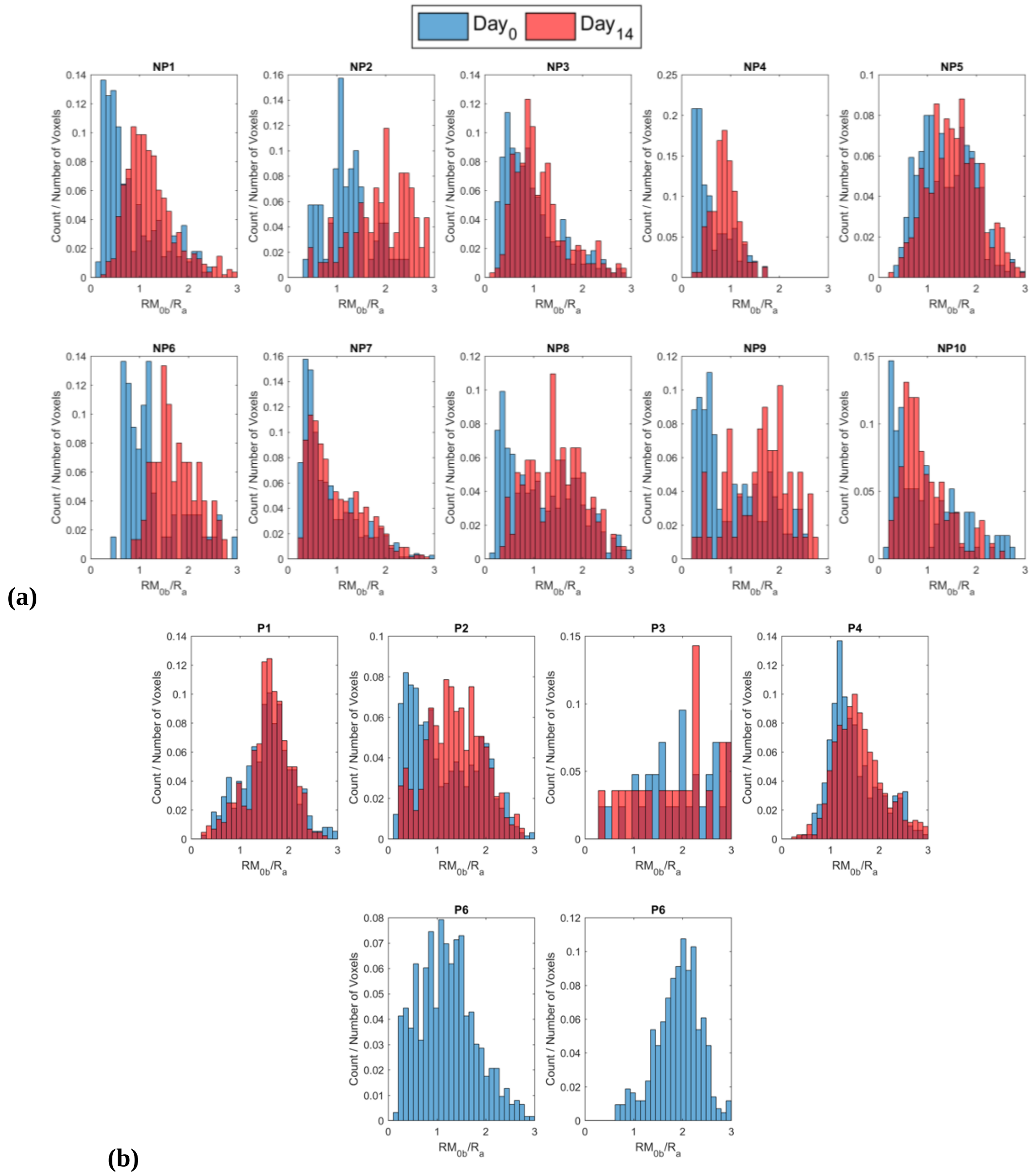

Figure S-2 Histogram distribution of RM_{0b}/R_a for each patients (P:progressor, NP:Non-progressor) at Day_0 and Day_{14} scans for ROI type I. Histogram bin counts are normalized with respect to the total number of voxels in the ROI to account for having different number of voxels in the ROIs.

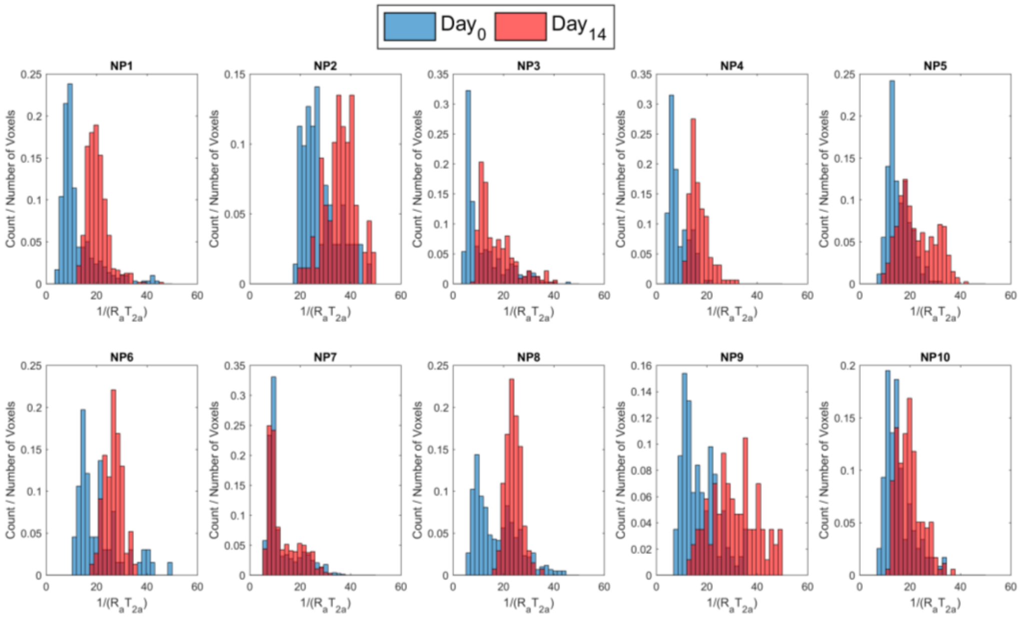

(a)

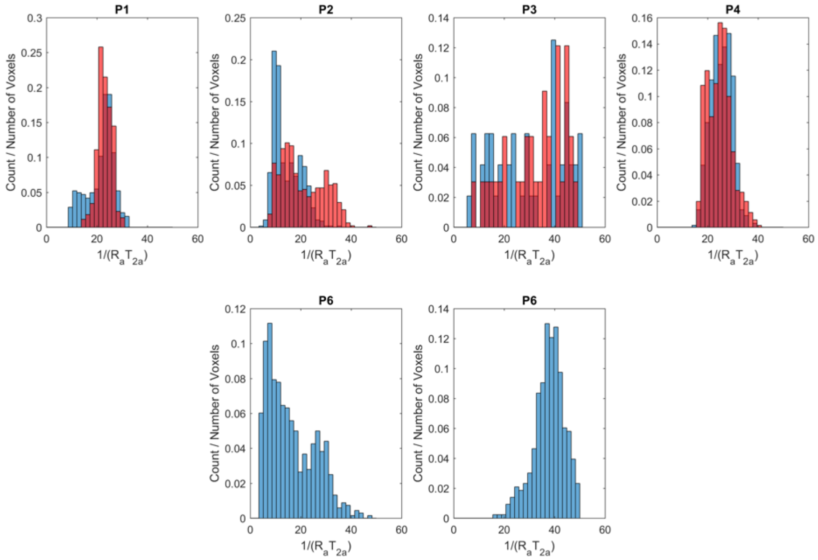

(b)

Figure S-3 Histogram distribution of $1/(R_a T_{2a})$ for each patients (P:progressor, NP:Non-progressor) for Day$_0$ and Day$_{14}$ scans for ROI type I. Histogram bin counts are normalized with respect to the total number of voxels in the ROI to account for having different number of voxels in the ROIs.

Table S-1 Histogram-based (Mean, median, Kurtosis, Skewness) quantitative MT parameter values for progressors (P) and non-progressors (NP) over ROI type I was calculated. Then mean ± standard deviation of the metric across all subjects in each cohort is reported.

		RM_{0b}/R_a				$1/(R_aT_{2a})$			
		Mean	Median	Skewness	Kurtosis	Mean	Median	Skewness	Kurtosis
Day_0	NP	**1.06±0.24****	**0.90±0.29****	**1.02±0.31****	4.0 ± 1.4	**16.2 ± 5.5 ***	**14.1 ± 5.8 ***	**1.15±0.43*****	3.9 ± 1.5
	P	**1.64 ± 0.48**	**1.60 ± 0.55**	**0.34 ± 0.40**	3.0 ± 0.6	**24.3 ± 8.8**	**24.0 ± 9.8**	**0.04 ± 0.65**	2.8 ± 0.9
Day_{14}	NP	1.38 ± 0.39	1.31 ± 0.44	0.67 ± 0.49	3.7 ± 1.2	22.7 ± 7.0	21.7 ± 7.9	0.64 ± 0.58	3.5 ± 1.4
	P	1.77 ± 0.50	1.74 ± 0.48	0.05 ± 0.51	2.9 ± 0.7	25.3 ± 5.1	25.5 ± 7.1	0.03 ± 0.48	2.6 ± 0.6
Day_{28}	NP	1.43 ± 0.26	1.33 ± 0.32	0.59 ± 0.39	3.1 ± 1.1	22.2 ± 5.9	21.6 ± 7.3	0.74 ± 0.74	3.8 ± 1.5
	P	1.71 ± 0.18	1.72 ± 0.19	0.19 ± 0.32	3.3 ± 0.3	24.3 ± 3.4	23.7 ± 2.9	0.47 ± 0.47	2.8 ± 0.9
Day_{70}	NP	1.56 ± 0.40	1.46 ± 0.51	0.76 ± 0.67	3.7 ± 0.8	24.1 ± 4.0	22.9 ± 4.8	0.84 ± 0.82	4.5 ± 1.5
	P	1.68 ± 0.06	1.61 ± 0.08	0.74 ± 0.52	4.8 ± 1.4	26.0± 3.6	24.6 ± 3.6	0.97 ± 0.63	4.1 ± 2.6

P: progressor, NP: non-progressor, *: $p<0.05$, **: $p<0.01$

Table S-2 Histogram-based (Mean, median, Kurtosis, Skewness) quantitative MT parameter values for progressors (P) and non-progressors (NP) over ROI type II was calculated. Then mean ± standard deviation of the metric across all subjects in each cohort is reported.

		RM_{0b}/R_a				$1/(R_aT_{2a})$			
		Mean	Median	Skewness	Kurtosis	Mean	Median	Skewness	Kurtosis
Day_0	NP	**1.06±0.24****	**0.90±0.29****	**1.02±0.31****	4.0 ± 1.4	**16.2 ± 5.5 ***	**14.1 ± 5.8 ***	**1.15±0.43*****	3.9 ± 1.5
	P	**1.64 ± 0.48**	**1.60 ± 0.55**	**0.34 ± 0.40**	3.0 ± 0.6	**24.3 ± 8.8**	**24.0 ± 9.8**	**0.04 ± 0.65**	2.8 ± 0.9
Day_{14}	NP	1.55 ± 0.36	1.51 ± 0.46	0.35 ± 0.67	3.2 ± 0.9	23.8 ± 7.0	23.0 ± 8.6	0.36 ±0.70	3.2 ± 0.8
	P	1.93 ± 0.65	1.95 ± 0.71	-0.11 ± 0.60	2.8 ± 0.4	26.2 ± 4.7	25.7 ± 5.1	0.45 ± 0.44	4.2 ± 3.6
Day_{28}	NP	1.68 ± 0.27	1.68 ±0.34	0.51 ± 0.75	4.0 ± 1.6	23.0 ± 4.9	22.7 ± 5.9	0.66 ± 0.85	4.3 ± 2.5
	P	1.85 ± 0.23	1.88 ± 0.28	0.04 ± 0.46	3.6 ± 0.7	25.3 ± 2.7	25.1 ± 1.8	0.35 ± 0.34	3.1 ± 0.5
Day_{70}	NP	1.73 ± 0.35	1.69 ±0.40	0.43 ± 0.57	3.7 ± 1.3	23.7 ± 3.5	23.3 ± 3.9	0.41 ± 0.80	4.2 ± 1.2
	P	1.70 ± 0.11	1.63 ± 0.13	0.08 ± 0.17	4.6 ± 0.3	25.8 ± 2.3	25.0 ± 2.3	0.71 ± 0.43	3.7 ± 1.7

P: progressor, NP: non-progressor, *: $p<0.05$, **: $p<0.01$
